# Genome‐wide virus‐integration analysis reveals a common insertional mechanism of HPV, HBV and EBV

**DOI:** 10.1002/ctm2.971

**Published:** 2022-08-15

**Authors:** Rui Tian, Yuyan Wang, Weiping Li, Zifeng Cui, Ting Pan, Zhuang Jin, Zhaoyue Huang, Lifang Li, Bin Lang, Jian Wu, Hongxian Xie, Yiqin Lu, Xun Tian, Zheng Hu

**Affiliations:** ^1^ Department of Obstetrics and Gynecology, Academician expert workstation, the Central Hospital of Wuhan, Tongji Medical College Huazhong University of Science and Technology Wuhan Hubei 430014 China; ^2^ Center of Cervical Cancer Precision Prevention and Treatment Sun Yat‐sen University Nanchang Research Institution Nanchang Jiangxi 330200 China; ^3^ Department of Obstetrics and Gynecology, The First Affiliated Hospital Sun Yat‐sen University Guangzhou Guangdong 510080 China; ^4^ Department of Obstetrics and Gynecology General Hospital of People's Liberation Army Beijing 100039 China; ^5^ Institute of Human Virology, Key Laboratory of Tropical Disease Control of Ministry of Education, Zhongshan School of Medicine Sun Yat‐sen University Guangzhou Guangdong 510080 China; ^6^ School of Health Sciences and Sports Macao Polytechnic Institute Macao 999078 China; ^7^ MyGenostics Inc Beijing 101300 China; ^8^ Generulor Company Bio‐X Lab Zhuhai Guangdong 519000 China; ^9^ Department of Gynecology, Dongzhimen Hospital Beijing University of Chinese Medicine Beijing 100700 China

Dear Editor,

Human papillomavirus (HPV), hepatitis B virus (HBV) and Epstein–Barr virus (EBV) are the three most oncogenic DNA viruses, contributing to 15 different types of cancer.^1^ Although these viruses differ in many aspects, one common key step is the integration of their DNA into the human genome, which could potentially promote carcinogenesis.^2^, ^3^, ^4^ In this study, we developed and performed a novel pipeline (Figures S1–S8, Supplementary Notes 1–3 and Table S1) named viral integration pathway analysis (VIPA) to elucidate the integration mechanism shared by HPV, HBV and EBV, thus gaining a deeper understanding towards the virus‐induced carcinogenesis and the corresponding anticancer therapies.

First, we conducted HPV capture sequencing and identified 1002 HPV integration breakpoints in 24.8% (225/910) non‐cancer HPV infection samples, 588 breakpoints in 38.0% (125/329) cervical precancer samples and 1597 breakpoints in 69.0% (158/227) cancer samples (Figure 1A). The total integration sample proportion was 34.7% (508/1466), and the average integration breakpoints were 6.27 per sample. We observed 24 recurrent integration hotspots (integration positions located within the 500‐kb downstream/ upstream of the gene, *n* ≥ 5) in our dataset (Figure 1A). Among them, 10 integration hotspots were previously reported, and 14 HPV integration hotspot genes were newly identified (Table S2).

**FIGURE 1:**
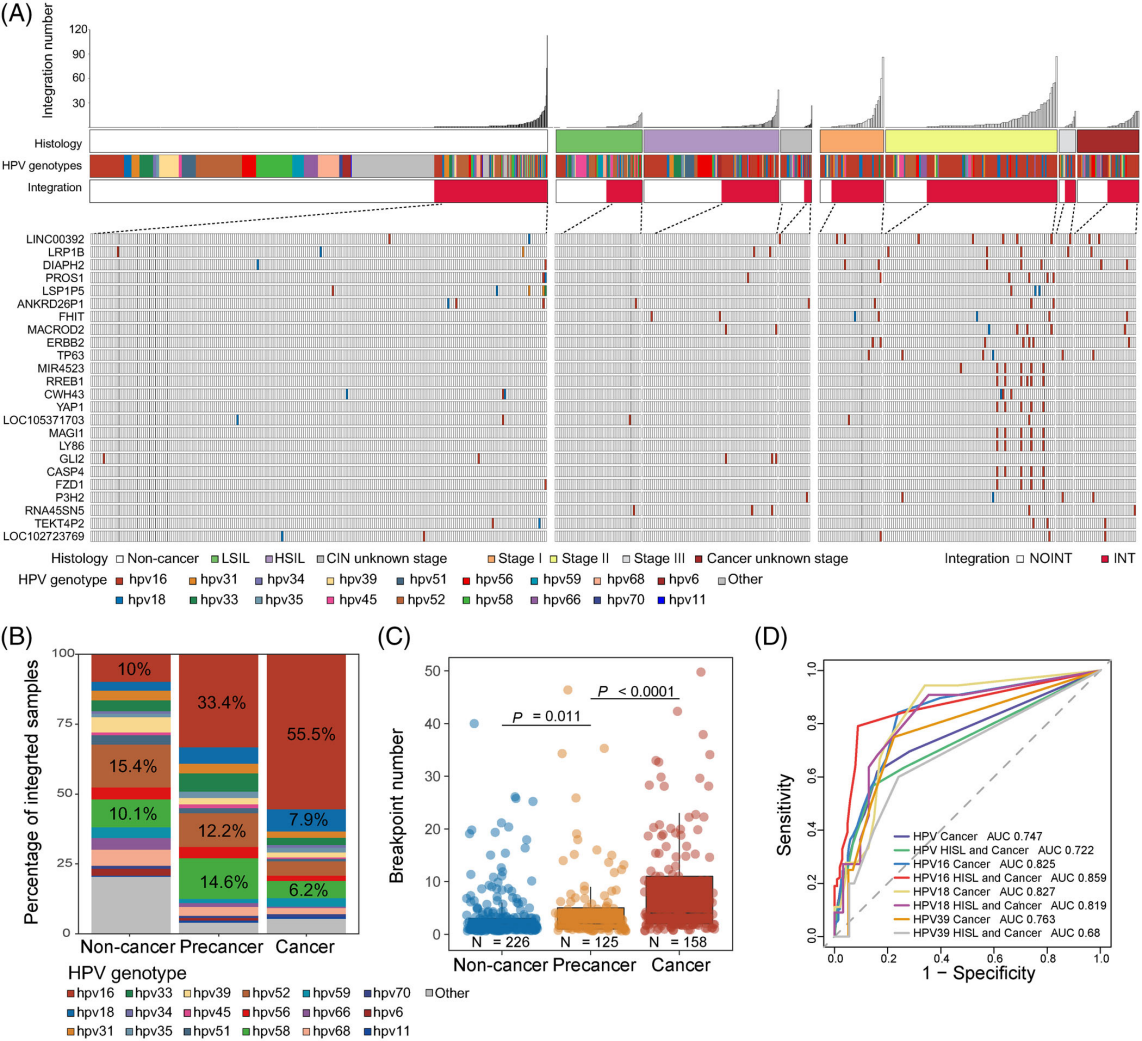
Theintegration landscape of new human papillomavirus (HPV) positive samples. (A) The landscape of our new HPV positive samples, including 910 HPV infection samples, 329 cervical precancer samples and 227 cervical cancer samples. The integration sample proportions were 24.8% for non‐cancer HPV infection (225/910), 38.0% for cervical precancer (125/329) and 69.0% for cancer stages (158/227). Among previous HPV‐integrated samples, there were 24 recurrent integration genes (*n* ≥ 5 samples) were shown; (B) the distribution of integrated HPV strains in three cervical disease stages. The percentages of top three HPV strains were marked; (C) the average integration events among non‐cancer infection, cervical precancer and cancer. Adjusted *p* values were calculated by *Wilcox* test; (D) the ROC of different HPV strains’ average integration events to predict stages more severe than high‐grade squamous intraepithelial lesion (HSIL) (HSIL and cancer) or cancer

Next, we found that the distribution of HPV integration strains and status in non‐cancer HPV infection, cervical precancer and cancer samples were different (Figure 1B,C). Specifically, HPV16 integration percentage was only 10% (ranked third) in non‐cancer samples but increased to 33.4% (ranked first) in precancer and 55.5% (ranked first) in cancer samples. HPV18 integration percentage was only 3.1% in non‐cancer samples, and 5.8% in precancer samples, and rose to 7.9% (ranked second) in cancer samples.

The average integration events for non‐cancer infection were 4.4, for cervical precancer were 4.7 and for cancer samples were 10.1, indicating that HPV integration increased along with the disease progression (non‐cancer vs. precancer, *p* = .011; precancer vs. cancer, *p* < .0001; *Wilcox* test, False Discovery Rate corrected) and may serve as an early warning biomarker of carcinogenesis (Figure 1C). When applying the average integration events to predict clinical outcomes, the results showed that we could distinguish high‐grade squamous intraepithelial lesion (HSIL)± (including HSIL and Cancer) with an AUC of .722. Further, we found that HPV16 held best prediction performance towards HSIL± with the AUC of .859. Similarly, HPV18 shared comparable prediction performance towards HSIL± with the AUC of .819 (Figure 1D).

Further, motivated by the aim of finding common integration features among HPV, HBV and EBV, we collected the capture sequencing data of the three viruses. Together, we detected 4390 integration breakpoints for HPV, 4010 integration breakpoints for HBV and 174 integration breakpoints for EBV (Tables S3–S5). Intriguingly, 21 integration genes were shared by all three viruses (Table S6), indicating the potential roles of these genomic loci in oncogenic viruses‐related cancers.

Next, we explored the viral integration patterns using identified human–viral junctional sequences (defined by ≥30‐bp human and viral sequences at the integration sites) from expanded integration datasets (Table S7 and Supplementary Notes 4 and 5). Previous studies have indicated that the integrations of three viruses were mediated by microhomology (MH)^4^, ^5^, ^6^, ^7^ (Figure S9). However, it is not clear how the lateral microhomologies (defined as microhomologies with short‐distance from the junction sites) mediate the integration process (Figure 2A–C). Inspired by the new understandings towards alternative end‐joining,^8^, ^9^ we speculated that synthesis‐dependent end‐joining (SD‐EJ) pathway may participate in the integration process to generate multiple types of breakpoints (Figure S10), including apparent blunt joining (Figure 2A), short insertion (Figure 2B) and junctional microhomologies (Figure 2C). We validated integration structures using the nanopore sequencing of Ca Ski DNA and Sanger sequencing of Ca Ski, HepG2.2.15 and Raji (Figures S11 and S12).

**FIGURE 2:**
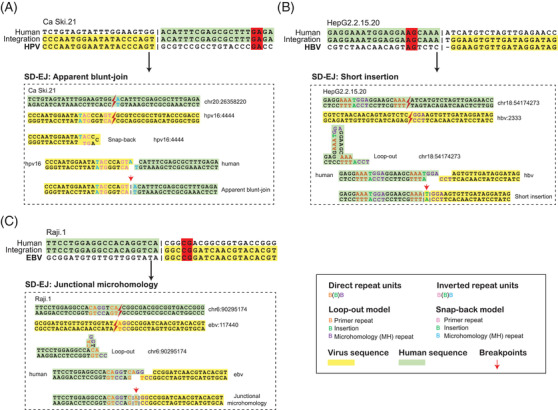
The illustration of synthesis‐dependent microhomology‐mediated end‐joining (SD‐EJ) integration pathway. Examples of SD‐EJ proposal model in the integration process of Ca Ski.21 breakpoint (A), HepG2.2.15.20 (B) and Raji.1 (C), which had lateral microhomologies

We analysed the roles of SD‐EJ using computational simulation (Figure S13) in 4341 human–HPV junctional sequences (Table S3), 4010 human–HBV junctional sequences (Table S4) and 169 human–EBV junctional sequences (Table S5). We found that SD‐EJ was significantly enriched for all three viruses (Figure 3A).

**FIGURE 3:**
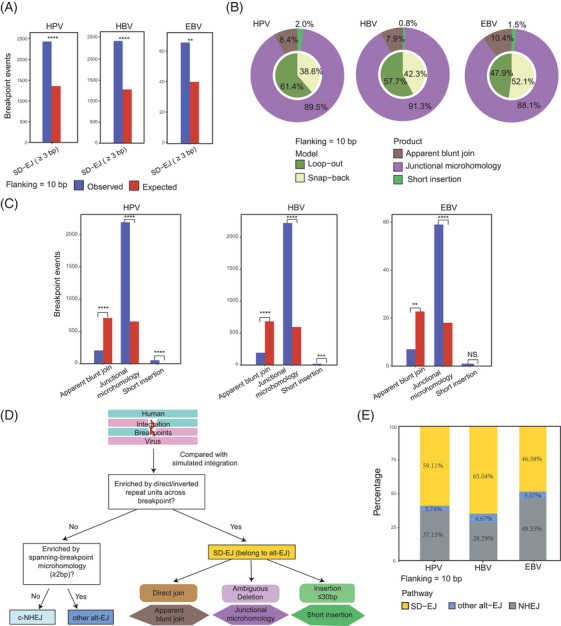
The synthesis‐dependent end‐joining (SD‐EJ) pathways in human papillomavirus (HPV), hepatitis B virus (HBV) and Epstein–Barr virus (EBV) integration datasets. (A) The comparison of integration events with SD‐EJ repeats (≥3 bp) between observed (actual) and expected (simulated) groups within 10‐bp flanking length. The previous *p* values were calculated by *Fisher's* exact test. *p < .05, **p < .01, ***p < .001, ****p < .0001; (B) the composition of two models (loop‐out and snap‐back) and three products (apparent blunt join, junctional microhomology and short insertion) of SD‐EJ integration events in HPV, HBV and EBV datasets within 10‐bp flanking length; (C) the comparison of three products (apparent blunt join, junctional microhomology and short insertion) between observed and expected groups within 10‐bp flanking length for HPV, HBV, EBV datasets. The previous *p* values were calculated by *Fisher's* exact test. *p < .05, **p < .01, ***p < .001, ****p < .0001. (D) The workflow details of further classification of integration pathways; (E) the proportions of SD‐EJ, other alt‐EJ and c‐NHEJ pathways in HPV, HBV and EBV datasets within 10‐bp flanking length

Then, the repair models and products of SD‐EJ were further analysed (Figure 3B). The proportions of loop‐out model were 47.9%–61.4% (HPV: 61.4%; HBV: 57.7% and EBV: 47.9%), whereas those of snap‐backs were 38.8%–52.1% (HPV: 38.8%; HBV: 42.3% and EBV: 52.1%). For repair products, junctional MH was the major type, accounting for 89.5% HPV, 91.3% HBV and 88.1% EBV SD‐EJ integration events, followed by apparent blunt join (HPV: 8.4%; HBV: 7.9% and EBV: 10.4%) and short insertion (HPV: 2.0%; HBV: .8% and EBV: 1.5%). The occurrence of junctional MH was significantly higher in the observed group than that in the expected group (Figure 3C, Supplementary Note 6). Conversely, the occurrence of apparent blunt join was significantly lower in the observed group than in the expected group. Of note, the significant enrichment of short insertion was observed in HPV and HBV datasets, whereas there was no significant difference of short insertion between EBV's observed and expected groups (*n* = 1 vs. *n* = .14, *p* = 1, *Fisher's* exact test) due to relatively small dataset (Figure 3C, Supplementary Note 6).

Finally, we classified integration pathways of each dsDNA virus breakpoint into three categories: (i) SD‐EJ pathway with SD‐EJ structures, followed by (ii) other alt‐EJ pathway with microhomologies overhangs and otherwise (iii) NHEJ pathway without the previous two signatures (Figure 3D). In 10‐bp flanking length, we observed the percentages of SD‐EJ pathway were 59.11% for HPV, 65.04% for HBV and 48.38% for EBV, whereas those of unclassified NHEJs were 37.15% for HPV, 28.29% for HBV and 48.55% for EBV (Figure 3E). The previous data suggested that SD‐EJ repair pathway may play an important role in the integrations of three viruses into human genome.

Together, we report the largest genome‐wide landscape of HPV, HBV and EBV insertional mutageneses. We uncovered HPV, HBV and EBV to share the same common SD‐EJ integration mechanism. Based on our identified integration patterns and the biology features of three viruses, we proposed a new model of the integration process of HPV, HBV and EBV (Figure 4), providing insights into virus‐induced cancer.

**FIGURE 4:**
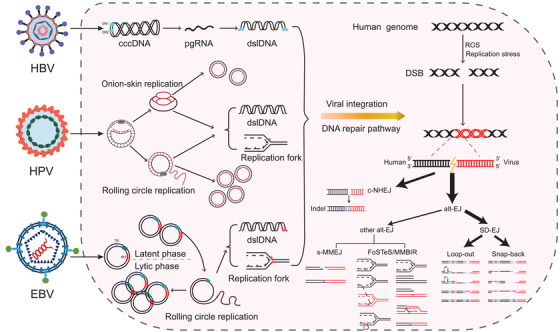
Model of DNA repair pathways involved in the integration of human papillomavirus (HPV), hepatitis B virus (HBV) and Epstein–Barr virus (EBV). Although viruses are replicated in different ways, their common feature is the production of large amounts of double‐stranded linear DNA (dslDNA) and replication forks. When the host cells encounter replication stresses or genetic insults (e.g. ROS), these replication products could serve as substrates of DNA repair pathway for fusion with double stranded DNA breaks (DSBs) generated from human genome, thereby promoting virus integration. Our data demonstrate that viral insertional events of HPV, HBV and EBV are mainly mediated via synthesis‐dependent end‐joining (SD‐EJ) DNA repair mechanism, followed by c‐NHEJ and other alt‐EJ (s‐MMEJ and FoSTeS) DNA repair mechanisms.

## FUNDING INFORMATION

This work was supported by the National Science and Technology Major Project of the Ministry of science and technology of China (Grant no. 2018ZX10301402); The National Natural Science Foundation of China (Grant no. 32171465 and 82102392); General Program of Natural Science Foundation of Guangdong Province of China (Grant no. 2021A1515012438); the National Postdoctoral Program for Innovative Talent (Grant no. BX20200398); the China Postdoctoral Science Foundation (Grant no. 2020M672995); Guangdong Basic and Applied Basic Research Foundation (Grant no. 2020A1515110170); the Major projects of Wuhan Municipal Health Commission (Grant no. WX19M02); the National Ten Thousand Plan‐Young Top Talents of China.

## CONFLICT OF INTEREST

The authors declare that they have no competing interests.

## Supporting information

Supplementary Note 1 The flow chart of VIPA
Supplementary Note 2 The performance of detecting virus integration sites in simulation data
Supplementary Note 3 The accuracy of indels calling at virus integration sites in simulation data
Supplementary Note 4 Study design and sample collection
Supplementary Note 5 Virus capture sequencing
Supplementary Note 6 Statistical analysis
Figure S1 The flow chart of VIPA
Figure S2 The performance of detecting virus integration sites in simulation data
Figure S3 The sensitivities and specificities of indels calling at junction sites by VIPA in simulated data
Figure S4 The VIPA validation in cell line model
Figure S5 The IGV image of eight nanopore reads supporting HPV16 integration sites at chr19:55307406
Figure S6 The Sanger sequencing results of all validated breakpoints in Ca Ski cell line
Figure S7 The Sanger sequencing results of all validated breakpoints in HepG2.2.15 cell line
Figure S8 The Sanger sequencing results of all validated breakpoints in Raji cell line
Figure S9 The MHs of human viral junctional sequences in other studies.
Figure S10 The core algorithms of SD‐EJ
Figure S11 The display of integration events with MHs structures (10‐bp flanking regions) in three cell lines
Figure S12 The statistics of integration events with SD‐EJ structures (10‐bp flanking regions) in three cell lines
Figure S13 The schematic of simulation methodology used for comparison

Table S1 The integration structures of Ca Ski validated by nanopore sequencing
Table S2 The 24 recurrent integration genes of new HPV samples
Table S3 Dataset of HPV integration events
Table S4 Dataset of HBV integration events
Table S5 Dataset of EBV integration events
Table S6 The common integration genes shared by HPV, HBV and EBV
Table S7 The virus capture sequencing data source of dsDNA viruses (soft‐clip reads ≥3)
Table S8 HPV Integration breakpoints per sample at each locus
Table S9 HBV Integration breakpoints per sample at each locus
Table S10 EBV Integration breakpoints per sample at each locus
Table S11 HPV breakpoints confirmed by PCR amplification and Sanger sequencing
Table S12 Characteristics of HPV infections women without cervical disease
