# Supplementary material for: Genome‐wide virus‐integration analysis reveals a common insertional mechanism of HPV, HBV and EBV
Source: Clin Transl Med. 2022 Aug 15;12(8):e971. doi: 10.1002/ctm2.971 (PMC9376973; doi:10.1002/ctm2.971)
Supplement: Supplementary file 1 — Supplementary Note 1 The flow chart of VIPA Supplementary Note 2 The performance of detecting virus integration sites in simulation data Supplementary Note 3 The accuracy of indels calling at virus integration sites in simulation data Supplementary Note 4 Study design and sample collection Supplementary Note 5 Virus capture sequencing Supplementary Note 6 Statistical analysis Figure S1 The flow chart of VIPA Figure S2 The performance of detecting virus integration sites in simulation data Figure S3 The sensitivities and specificities of indels calling at junction sites by VIPA in simulated data Figure S4 The VIPA validation in cell line model Figure S5 The IGV image of eight nanopore reads supporting HPV16 integration sites at chr19:55307406 Figure S6 The Sanger sequencing results of all validated breakpoints in Ca Ski cell line Figure S7 The Sanger sequencing results of all validated breakpoints in HepG2.2.15 cell line Figure S8 The Sanger sequencing results of all validated breakpoints in Raji cell line Figure S9 The MHs of human viral junctional sequences in other studies. Figure S10 The core algorithms of SD‐EJ Figure S11 The display of integration events with MHs structures (10‐bp flanking regions) in three cell lines Figure S12 The statistics of integration events with SD‐EJ structures (10‐bp flanking regions) in three cell lines Figure S13 The schematic of simulation methodology used for comparison [file CTM2-12-e971-s002.docx]

**Supplementary Note 1.**

**The flowchart of VIPA**

We developed Virus Integration Pathway Analysis (VIPA) platform to discover integration patterns of the human-viral junctional sequences. Generally, VIPA includes five modules (Figure S1): (1) scanning virus species and genotype, (2) detecting virus integration for specific virus genotype, (3) obtaining human-viral junctional sequences, (4) calculating the MHs around the junction positions and (5) calculating SD-EJ around the junction positions.

VIPA aligns FASTQ reads to the yielded reference genome combining the human genome (GRCh38) and the virus library genome, using BWA-MEM^1^. The pre-built virus library references including 183 HPV genomes were downloaded from PaVE database (<http://pave.niaid.nih.gov>), 11 references of HBV strains (Accession number: AB014381.1, AB032431.1, AB033554.1, AB036910.1, AB064310.1, AF090842.1, AY090454.1, M32138.1, NC_003977.2, X02763.1, X51970.1) and 2 EBV gnomes (Accession number: NC_007605.1 and NC_009334.1). The unique mapped sequence reads of certain virus genotype will be examined in two aspects, including the mapping proportion (≥ 50%) and similarity (≥ 80%) to exclude the non-specific alignment results. Then, the default thresholds (reads≥100, depth≥10x and coverage ≥50%) will be used to generate the final reported virus genotypes.

Then, FASTQ reads will be aligned to the new reference consisting of genomes of human (*H*) and a specific virus (*V*) genotype again, followed by removing duplication by Picard tool and SAMtools ^2^. Meanwhile, the separate alignment file will also be produced (mapped to *H* and *V,* respectively). VIPA calculated alignment times in *H* (marked as A) and *V* (marked as B) results and whether A or B is ≥ 1 according to the soft-clip definition (both human and virus DNA sequences are in the same read). Then VIPA extract potential soft-clip alignment results and categorized them into three situations (Fig.1B): a) Discordant reads; b) Soft-clip pair reads (collapsed into one read by “bwa -pemerge”) and c) Soft-clip oneend reads. Then, we collected soft-clip reads in b) and c) and mapped them using another bioinformatic alignment tool BLASTN ^3^ to the human and virus genome. The consistently aligned reads were kept as confident soft-clip reads and clustered according to the positions in human (in silico validation 1). VIPA required at least ≥ 3 soft-clip reads to report a viral integration event, which was verified in experiment data of cell line models. Considering of low frequent integration events or limited sequencing depth of virus, the criteria (minimum supporting soft-clip reads) is set as an option for users to choose according to their purposes. The integration events were then annotated by ANNOVAR ^4^ to illustrate the association with targeted genes.

VIPA then collected soft-clip reads and performed multiple alignment using MUSCLE ^5^, followed by yielding consensus sequence by EMBOSS ^6^ with the parameters “-plurality 0.00001”. The consensus sequence will be aligned by BLASTN and reported as human-viral junctional sequence if the expected human and viral locations were consistent (in silico validation 2). Then the human-viral junctional sequences will be used for MHs and SD-EJ calculation.

**Supplementary Note 2.**

**The performance of detecting virus integration sites in simulation data.**

It is important to guarantee the accuracy and sensitivity of virus integration sites identified by VIPA. Therefore, we compared the performance of VIPA with several software, including Virus-Clip^7^, ViFi^8^, VIcaller^9^ and SurVirus^10^, in a series of simulated data sets (Figure S2). The simulation involved full lengths of 4 sub-lineages for each virus genotype (HPV16, HBV type C and EBV type1), 2 paired-end read lengths (PE100 and PE150) and 4 depths (5 x, 10 x, 20 x and 30 x). Generally, 992 breakpoints for HPV16, 992 breakpoints for HBV type C and 682 breakpoints for EBV type 1 were simulated with virus insertion fragment of 500 - 1000 bp.

Based on the accurate integration sites in human, we summarized the sensitivity and the specificity values of each software. For all three viruses, VIPA exhibited high specificity (>99%) regardless of read depths and lengths while the sensitivities of VIPA were higher when read depths and lengths increasing and had comparable sensitivities with VIcaller and SurVirus when read depths ≥10x. ViFi had similar specificity level (over 99%) as VIPA while the sensitivities were lower than VIPA. In contrast, Virus-Clip had similar sensitivity level as VIPA while the specificities of Virus-Clip were not as good.  Taken together, above results demonstrated that VIPA is a valuable virus integration detection tool in regard to high accuracy and sensitivity.

**Supplementary Note 3.**

**The accuracy of indels calling at virus integration sites in simulation data.**

we chose the first 25% integration events of our 992 HPV, 992 HBV and 686 EBV simulated integration events, which were used in the evaluation of different software. The 248 HPV, 248 HBV and 171 EBV simulated integration events were further used to evaluate the accuracy of alignment at the junction, especially regarding the INDEL calling.

Specifically, the deletions at the junction (≥1 bp) shared by both human and virus genomes. There should be expected to detect 108, 109, 67 junctions with deletions for HPV, HBV and EBV. For insertions, we further randomly inserted 1-5 bp nucleotides into above simulated integration events. Then, we applied VIPA in 30x simulated NGS data of above integration events. Finally, we assessed the alignment at the junction by the stringent requirements of ≤3 bp mismatches around 20 bp regions around the junctions. The results showed, the sensitivities of deletions calling were 96.3% (104/108), 81.7% (89/109), 89.6% (60/67) for HPV, HBV and EBV, while the specificities were 98.1% (104/106), 92.7% (89/96), 90.9% (60/66) for HPV, HBV and EBV, respectively. For insertions calling, the sensitivities were 94.4% (234/248), 87.1% (216/248), 90.6% (155/169) for HPV, HBV and EBV, and the specificities were 99.2% (234/236), 96.9% (216/223), 96.9% (155/160) for HPV, HBV and EBV, respectively.

**Supplementary Note 4**

**Study design and sample collection.**

For HPV, we newly sequenced samples covering the whole spectrum of HPV-related cervical diseases, including 910 non-cancer infection (cervical exfoliated cells or cervical tissues from patients with HPV positive and histology negative test results). These samples are part of the sample collection from the Central Hospital of Wuhan, which were collected in Longyou county, Zhejiang province. All Patients signed the informed consent form, and underwent speculum examination by a gynecologist. According to instruction, samples of cervical exfoliate cells were collected using a cytology brush (Hologic, Bedford, MA) and stored in the tubes with preservation solution for the Thinprep cytology test (TCT, Hologic, Bedford, MA) and HPV DNA test (Cobas® 4800 Test, Roche Molecular Systems, Pleasanton, CA), respectively. All samples were collected during April to May of 2015. Cytology slides were read by two pathologists of our hospital and were reported according to the Bethesda 2014 classification. All women with positive HPV testing or abnormal cytology were referred to colposcopy. And two pathologists made the diagnosis based on 2014 WHO Classification of Tumor of the Female Genital Tract.

Besides, 130 samples (26 cervical precancer and 104 cancers) from SRA189003^11^ and 114 samples (25 non-cancer infection with the same definition as above, 44 cervical precancer and 45 cancers) from SRA315538^12^ studies were also involved.

For HBV, we included 426 tumor and 426 adjacent non-tumor samples from 426 HCC patients in SRA335342^13^ study.

For EBV, we involved 270 samples from SRP185895^14^ study, including 54 healthy saliva samples, 215 cancer samples (nasopharyngeal carcinoma: 179; Gastric carcinoma: 16; Hodgkin’s lymphoma: 11; NK/T cell lymphoma: 7; Burkitt’s lymphoma: 2) and 1 nasopharyngeal carcinoma cell line; Besides, the 404 samples from ERP001026^15^ study were also included with unavailable information.

**Supplementary Note 5**

**Virus capture sequencing**

The virus capture sequencing of Ca Ski, HepG2.2.15, Raji and expanded HPV-related cervical samples was performed by MyGenostics as previously described ^8, 16^. Virus capture sequencing of HPV-positive samples was performed using MyGenostics probes which designed against the full-length 17 HPV types (6, 11, 16, 18, 31, 33, 35, 39, 45, 52, 56, 58, 59, 66, 68, 69, and 82). Genomic DNA was sheared to 180–220 bp using the Covaris S220 System, followed by end repair, A-tailing, adaptor-ligation, and pre-PCR to construct the DNA library. Then, the library was hybridized with probes at 65 °C for 16 h and then washed with washing buffer 1, washing buffer 2 and 80% ethanol to remove un-targeted fragments. The eluted DNA was amplified by post-PCR to generate libraries for sequencing. The size and concentration of PCR products were assessed by Agilent 2100 and qPCR, respectively. Libraries were sequenced on the HiSeq X platform to generate 150 bp paired-end data and the sequencing data size was over 500 Mb per sample.

**Supplementary Note 6**

**Statistical analysis**

All tests were performed using R Programming Language 3.4.2 with a two-sided *P* value provided. As for gene integration events summary, at first, we summarized the integration counts at each gene locus in a single sample according to the definition of within the 500 kb flanking regions (Table S8-10). Further, we defined the gene integration events based on the integrated sample counts for each gene, which means multiple integrations into the same gene locus in multiple samples were calculated. Enrichment analysis between the observed and expected was calculated by *Fisher’s* exact test (*P* < 0.05 were considered statistically significant).

**
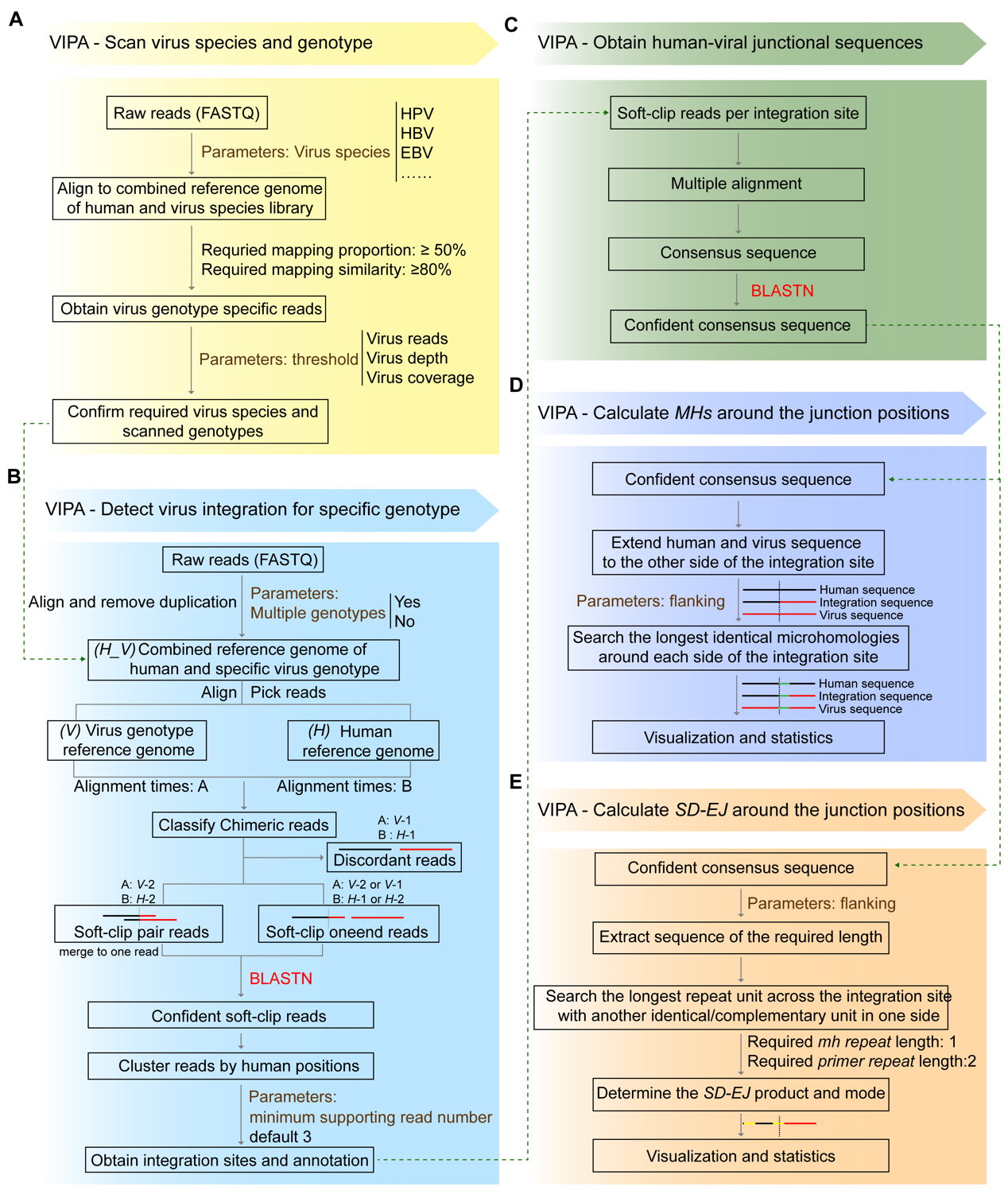
**

**Figure S1. The flowchart of VIPA.**

Five modules are involved in VIPA by order **(A)** scanning virus species and genotype, **(B)** detecting virus integration for specific virus genotype, **(C)** obtaining human-viral junctional sequences, **(D)** calculating the MHs around the junction positions by sequence analysis and **(E)** calculating SD-EJ around the junction positions by sequence analysis.


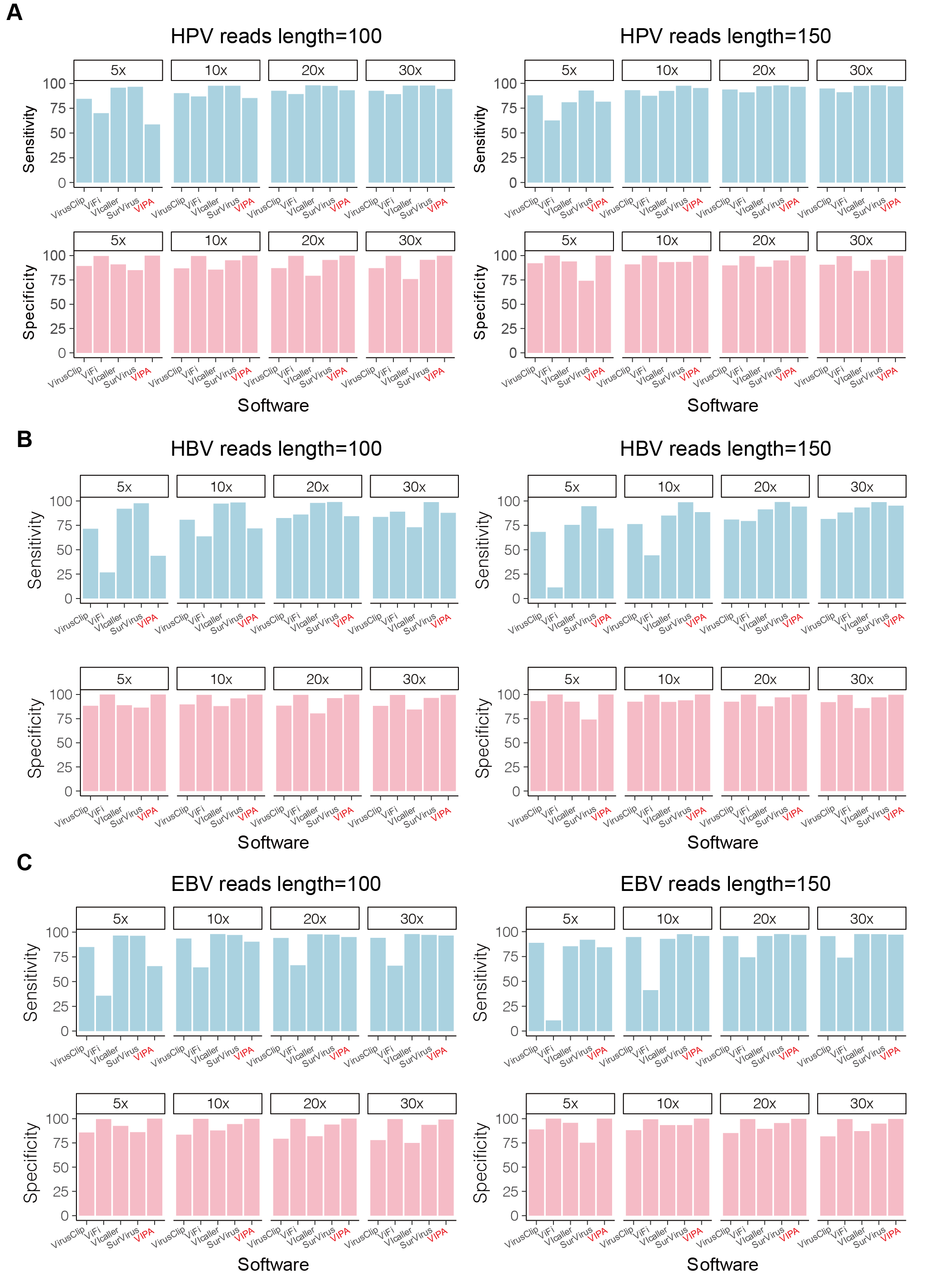


**Figure S2. The performance of detecting virus integration sites in simulation data.**

The performance comparison of Virus-Clip, ViFi, VIcaller, SurVirus and VIPA in simulated HPV **(A)**, HBV **(B)** and EBV **(C).** For each software, the sensitivity and specificity values of 4 reads depth (5x, 10x, 20x and 30x) were displayed in light blue and pink bars, respectively. The left and right panels showed the performance in PE100 and PE150 reads length modes.


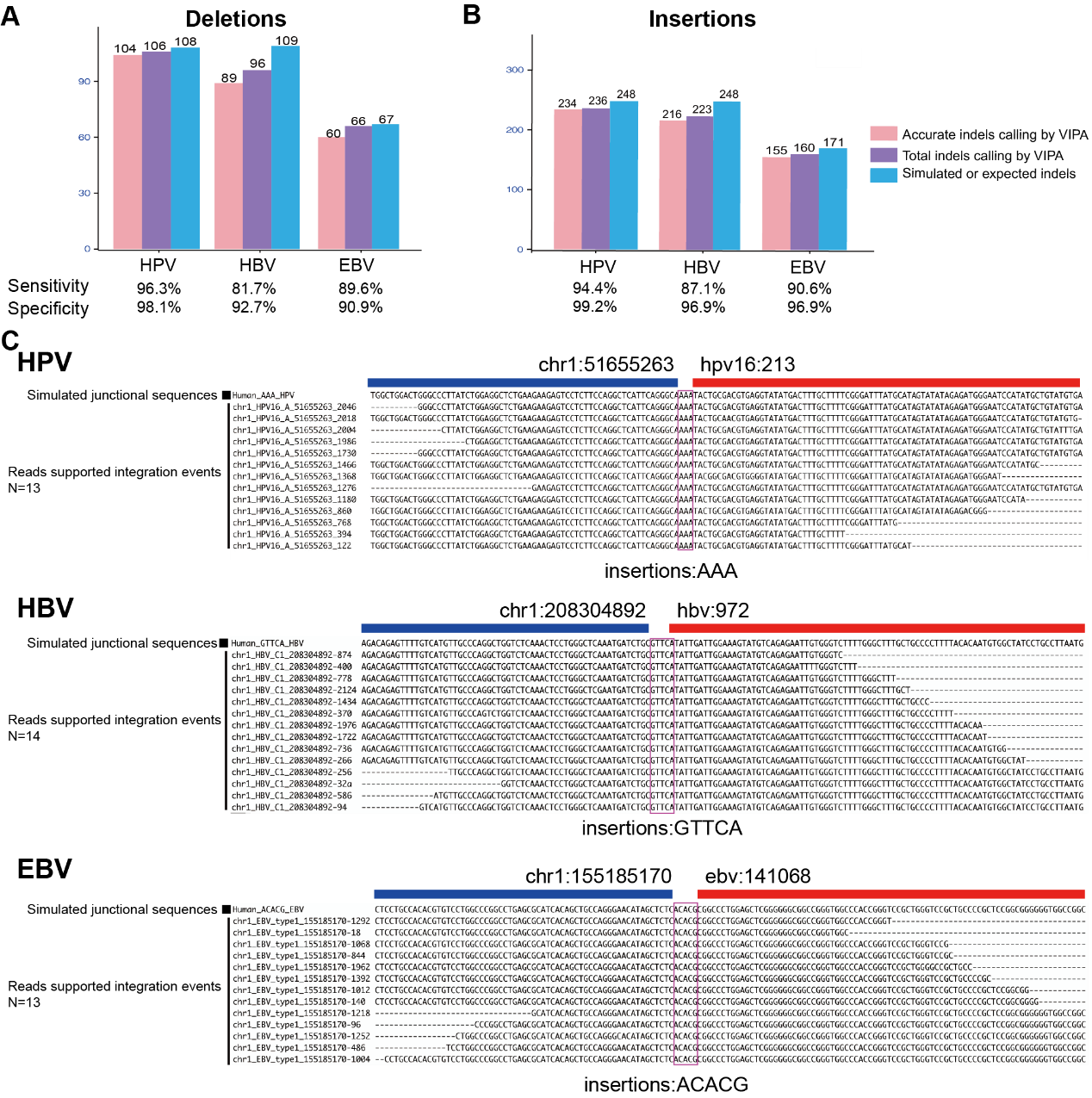


**Figure S3. The sensitivities and specificities of indels calling at junction sites by VIPA in simulated data.**

**(A)** The sensitivities and specificities of deletions calling at integration sites by VIPA **(B)** The sensitivities and specificities of insertions calling at integration sites by VIPA. **(C)** The multiple alignments of integration supported reads called by VIPA for HPV, HBV and EBV simulated data with insertions at junction sites.

**
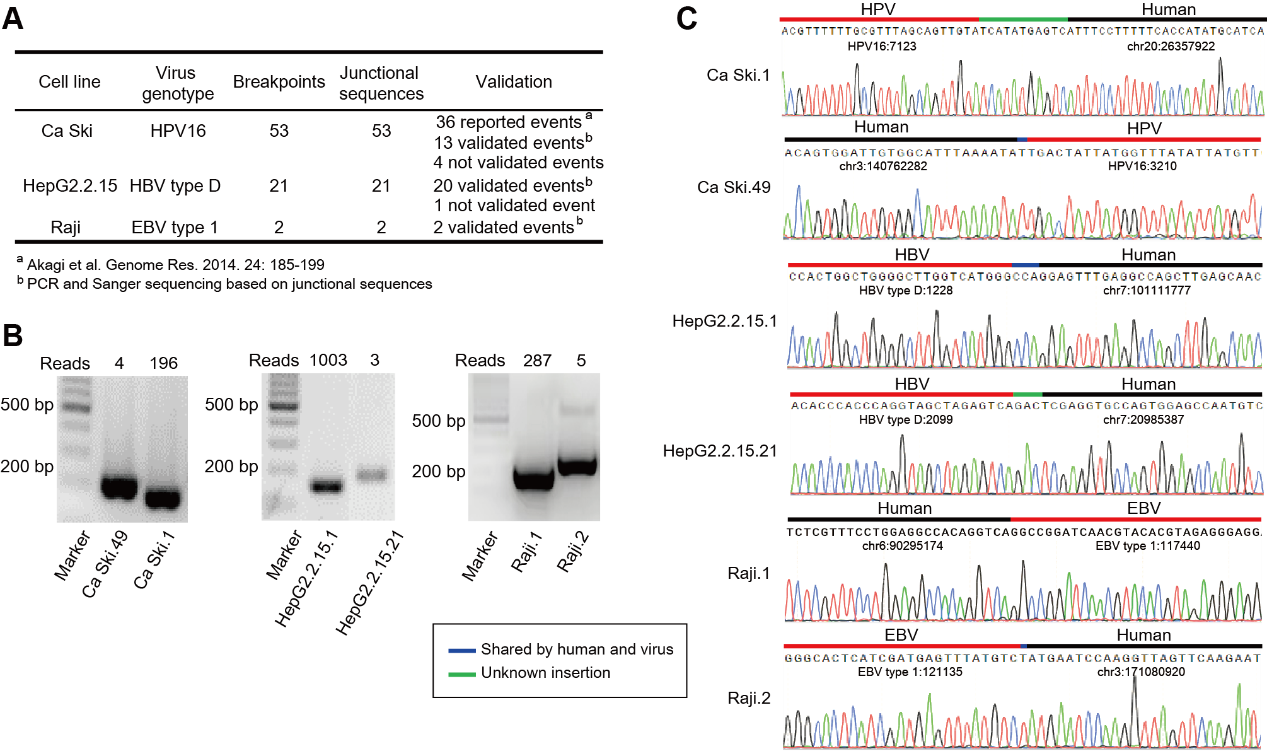
**

**Figure S4. The VIPA validation in cell line model.**

**(A)** The summary of integration sites detected by VIPA in capture sequencing data of three virus-related cell line models. **(B)** The gel image of PCR validated breakpoints detected by VIPA with maximum and minimum supporting soft-clip reads. **(C)** Sanger sequencing results of above PCR-validated breakpoints.


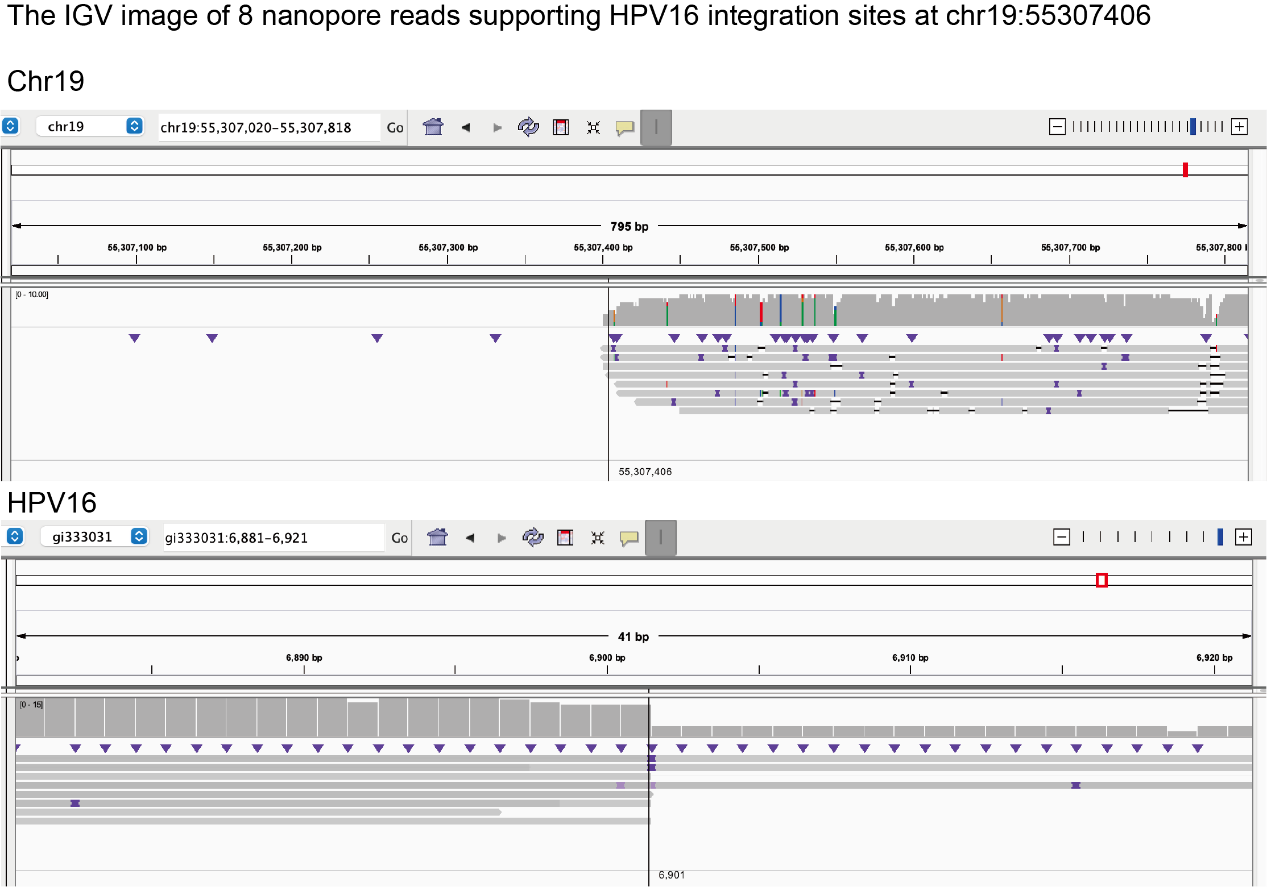


**Figure S5.** **The IGV image of 8 nanopore reads supporting HPV16 integration sites at chr19:55307406**


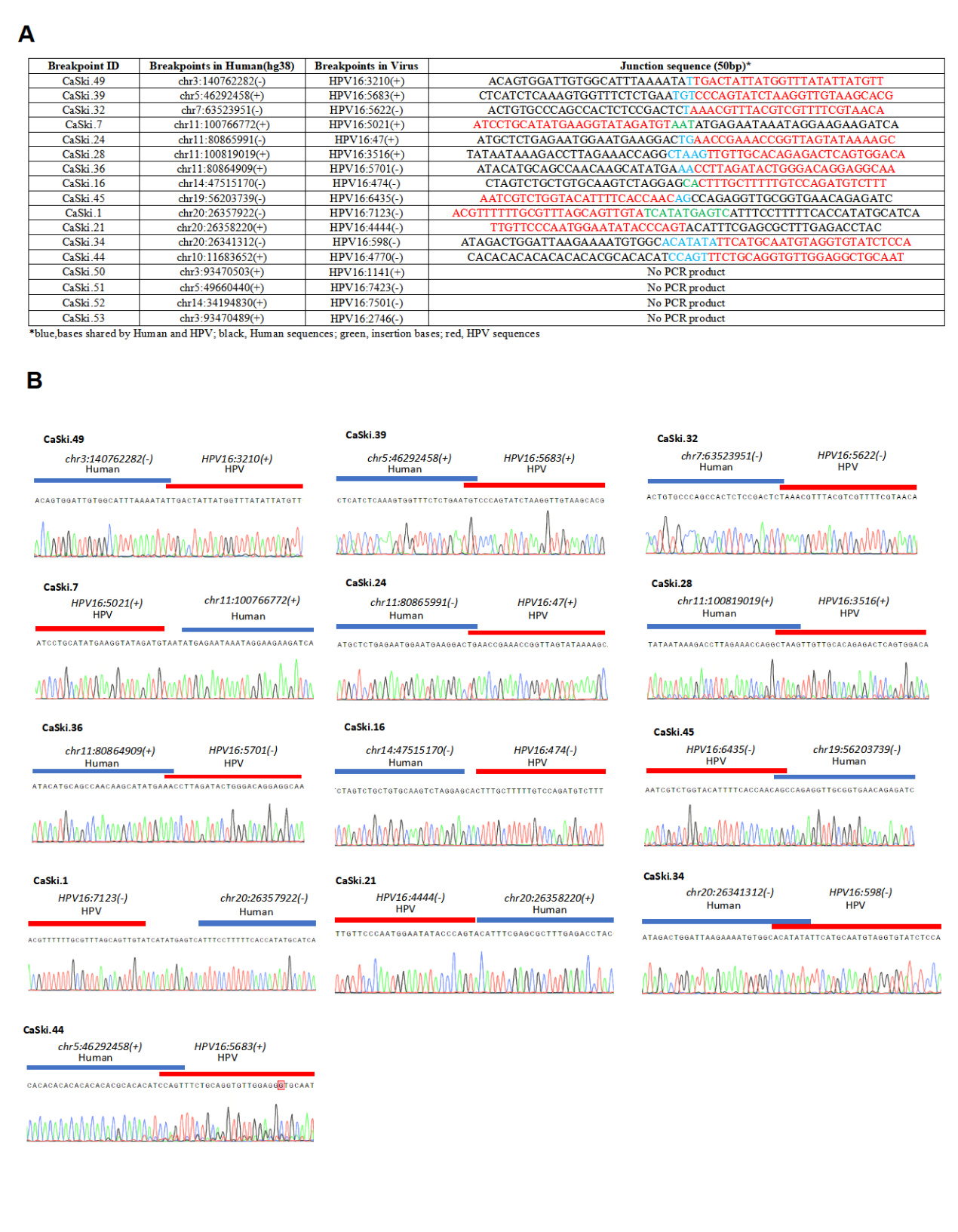


**Figure S6. The Sanger sequencing results of all validated breakpoints in Ca Ski cell line.**


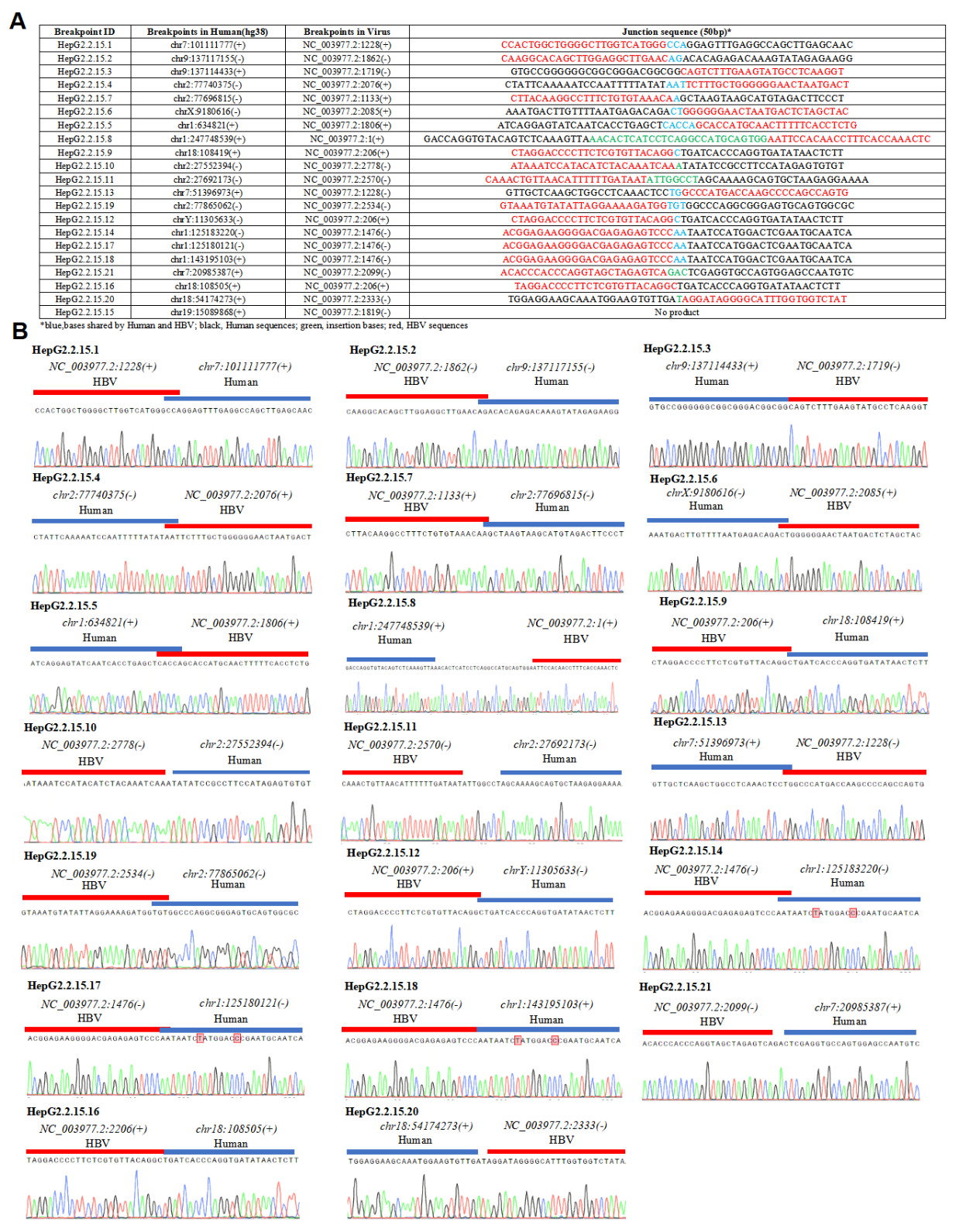


**Figure S7. The Sanger sequencing results of all validated breakpoints in HepG2.2.15 cell line.**


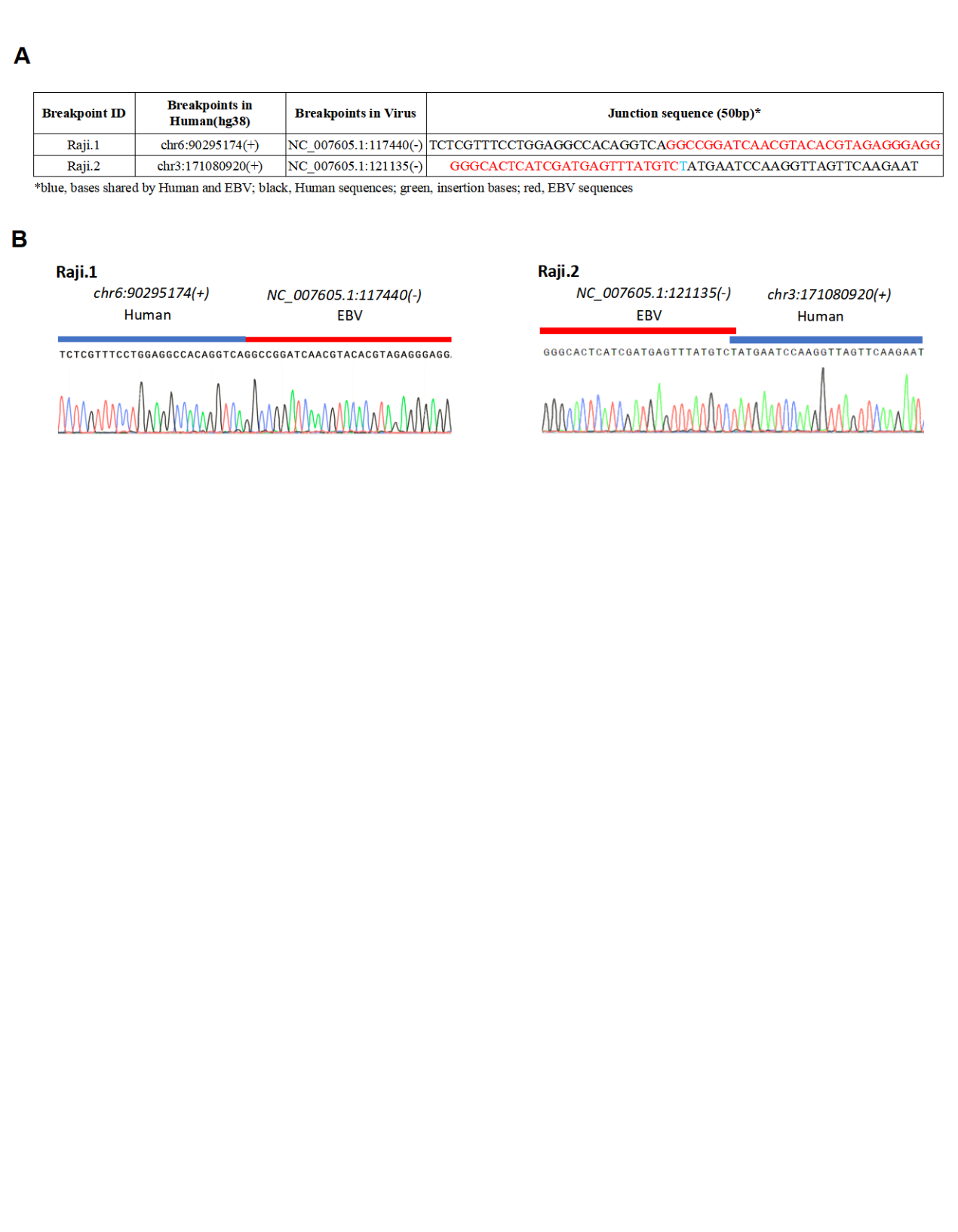


**Figure S8. The Sanger sequencing results of all validated breakpoints in Raji cell line.**


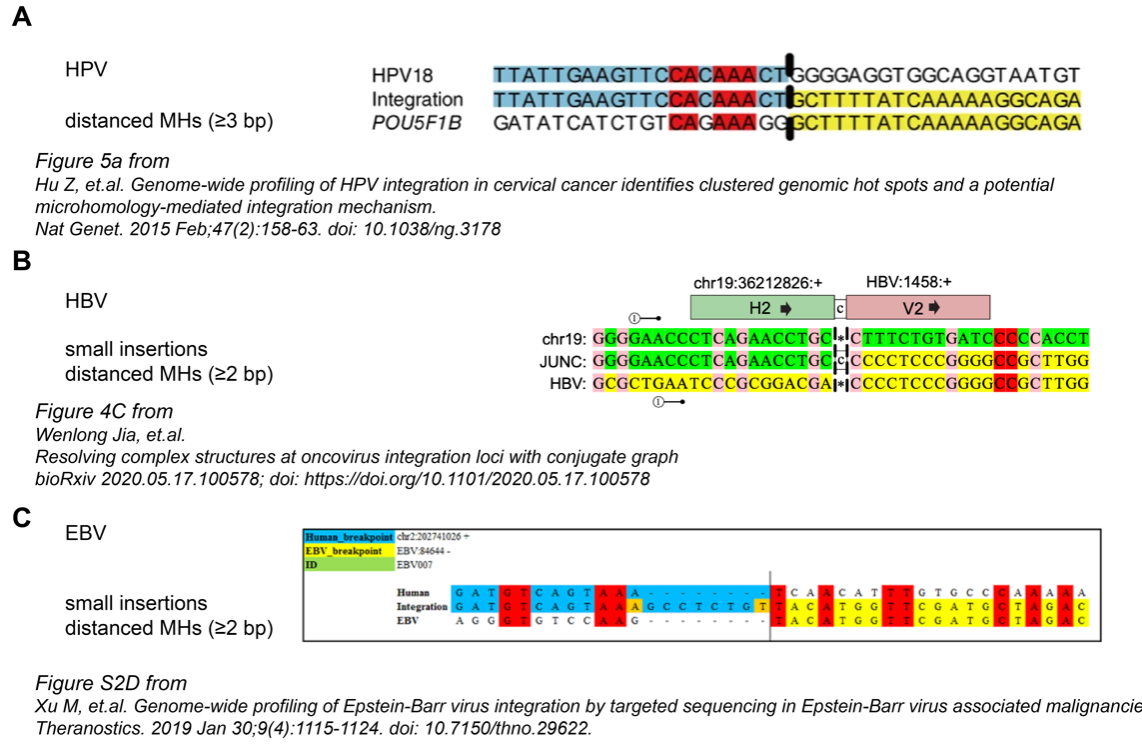


**Figure S9. The MHs of human viral junctional sequences in other studies.**

**
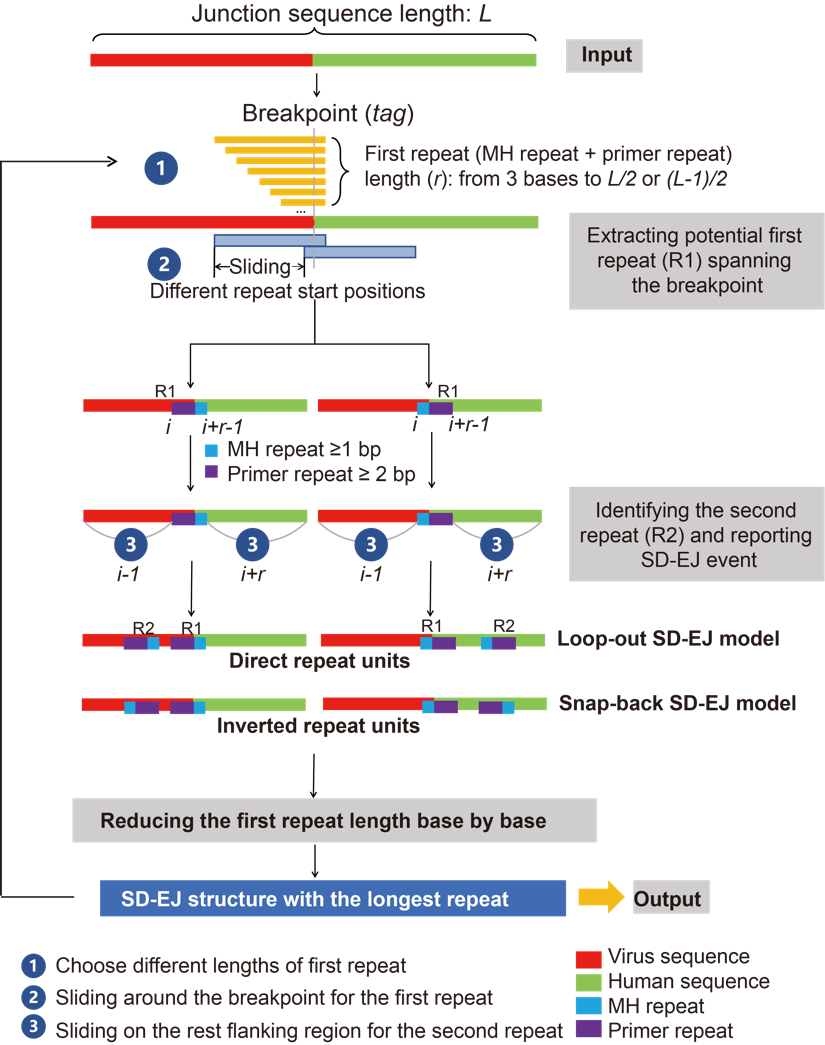
**

**Figure S10. The core algorithms of SD-EJ.**

**
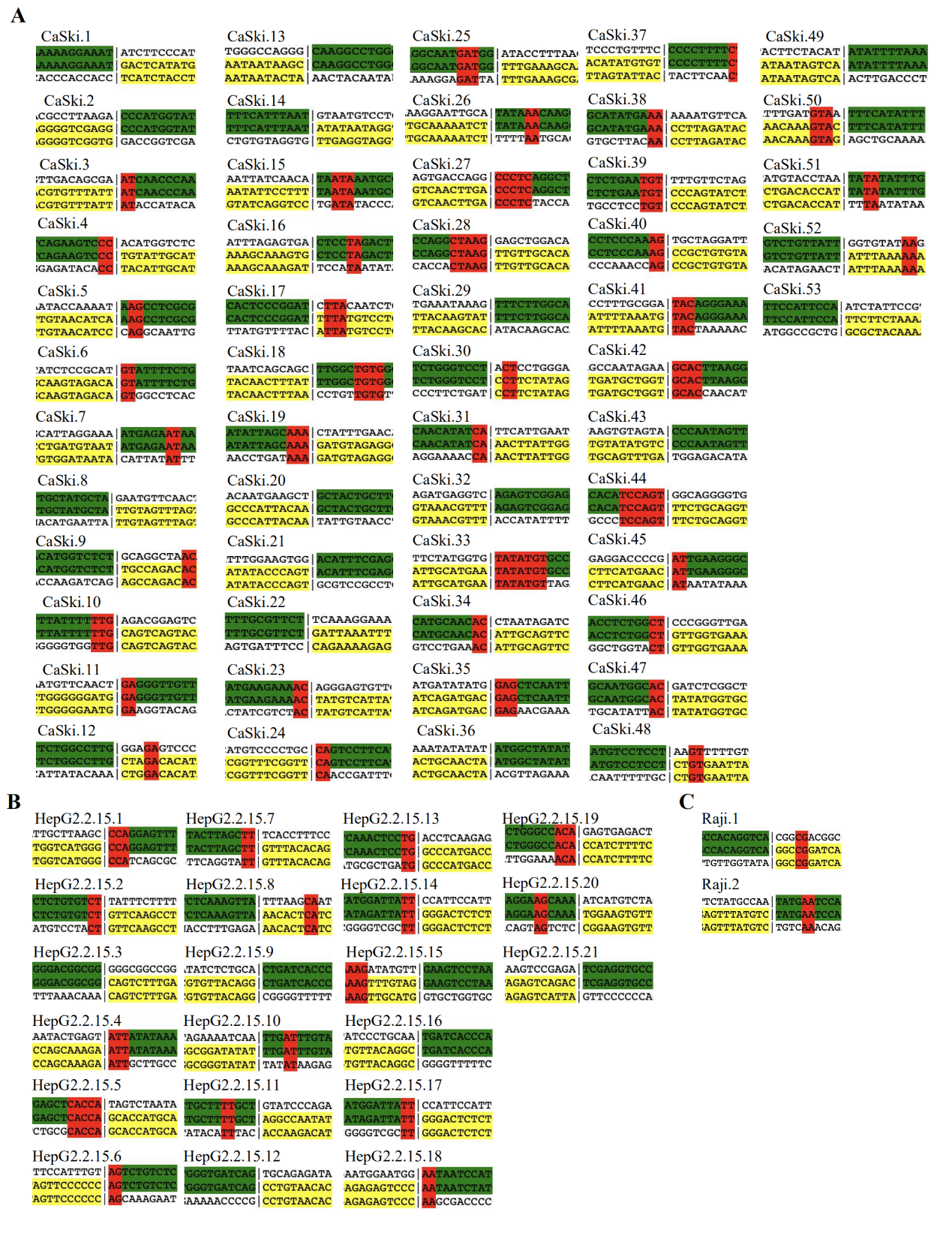
**

**Figure S11. The display of integration events with MHs structures (10 bp flanking regions) in three cell lines.**


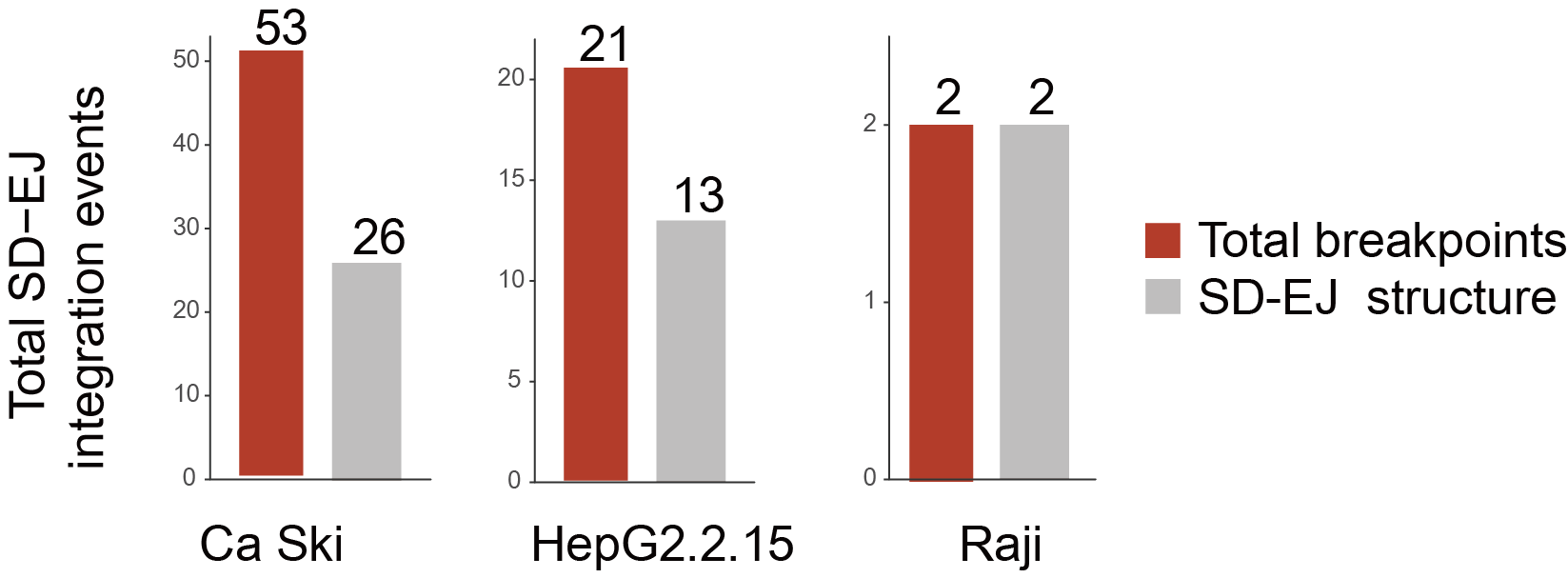


**Figure S12. The statistics of integration events with SD-EJ structures (10 bp flanking regions) in three cell lines.**


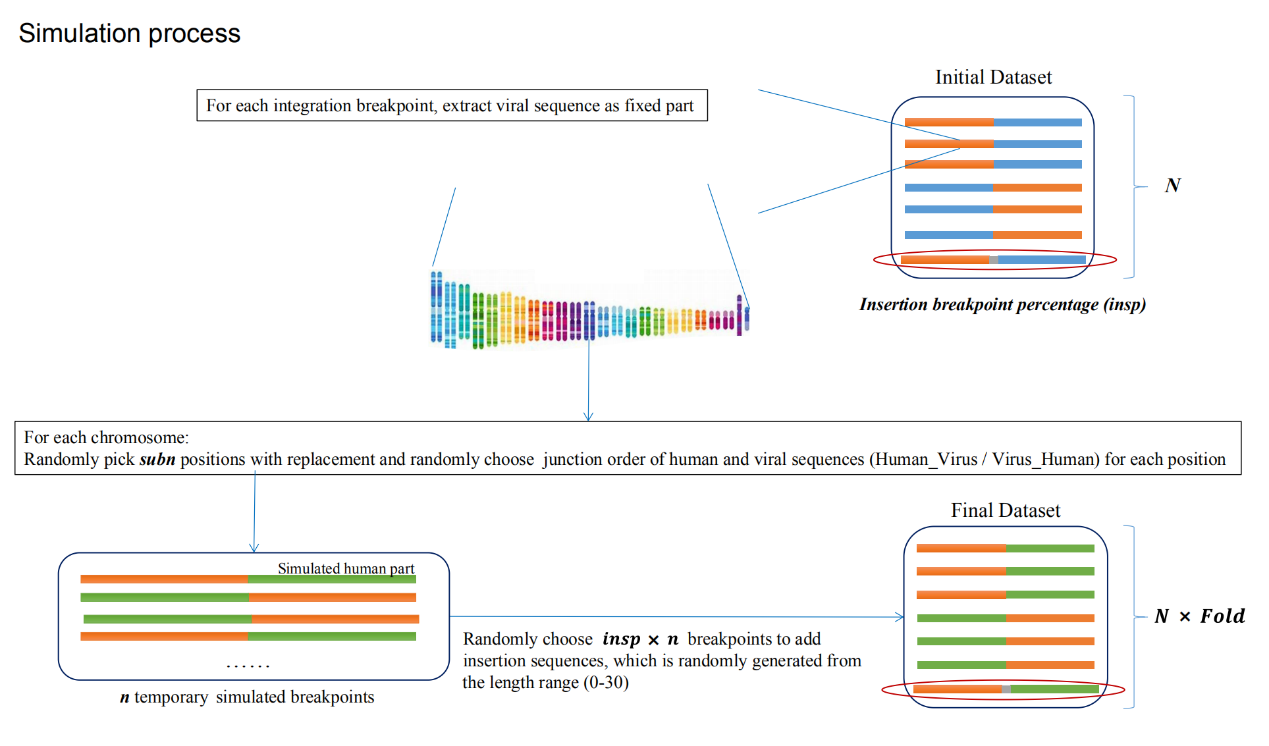


**Figure S13. The schematic of simulation methodology used for comparison.**

**References**

1. Li, H, and Durbin, R (2009). Fast and accurate short read alignment with Burrows-Wheeler transform. *Bioinformatics* **25**: 1754-1760.

2. Li, H, Handsaker, B, Wysoker, A, Fennell, T, Ruan, J, Homer, N, Marth, G, Abecasis, G, Durbin, R, and Genome Project Data Processing, S (2009). The Sequence Alignment/Map format and SAMtools. *Bioinformatics* **25**: 2078-2079.

3. Altschul, SF, Gish, W, Miller, W, Myers, EW, and Lipman, DJ (1990). Basic local alignment search tool. *J Mol Biol* **215**: 403-410.

4. Wang, K, Li, M, and Hakonarson, H (2010). ANNOVAR: functional annotation of genetic variants from high-throughput sequencing data. *Nucleic Acids Res* **38**: e164.

5. Edgar, RC (2004). MUSCLE: multiple sequence alignment with high accuracy and high throughput. *Nucleic Acids Res* **32**: 1792-1797.

6. Rice, P, Longden, I, and Bleasby, A (2000). EMBOSS: the European Molecular Biology Open Software Suite. *Trends Genet* **16**: 276-277.

7. Ho, DW, Sze, KM, and Ng, IO (2015). Virus-Clip: a fast and memory-efficient viral integration site detection tool at single-base resolution with annotation capability. *Oncotarget* **6**: 20959-20963.

8. Nguyen, ND, Deshpande, V, Luebeck, J, Mischel, PS, and Bafna, V (2018). ViFi: accurate detection of viral integration and mRNA fusion reveals indiscriminate and unregulated transcription in proximal genomic regions in cervical cancer. *Nucleic Acids Res* **46**: 3309-3325.

9. Chen, X, Kost, J, Sulovari, A, Wong, N, Liang, WS, Cao, J, and Li, D (2019). A virome-wide clonal integration analysis platform for discovering cancer viral etiology. *Genome Res* **29**: 819-830.

10. Rajaby, R, Zhou, Y, Meng, Y, Zeng, X, Li, G, Wu, P, and Sung, WK (2021). SurVirus: a repeat-aware virus integration caller. *Nucleic Acids Res* **49**: e33.

11. Hu, Z, Zhu, D, Wang, W, Li, W, Jia, W, Zeng, X, Ding, W, Yu, L, Wang, X, Wang, L*, et al.* (2015). Genome-wide profiling of HPV integration in cervical cancer identifies clustered genomic hot spots and a potential microhomology-mediated integration mechanism. *Nat Genet* **47**: 158-163.

12. Huang, J, Qian, Z, Gong, Y, Wang, Y, Guan, Y, Han, Y, Yi, X, Huang, W, Ji, L, Xu, J*, et al.* (2019). Comprehensive genomic variation profiling of cervical intraepithelial neoplasia and cervical cancer identifies potential targets for cervical cancer early warning. *J Med Genet* **56**: 186-194.

13. Zhao, LH, Liu, X, Yan, HX, Li, WY, Zeng, X, Yang, Y, Zhao, J, Liu, SP, Zhuang, XH, Lin, C*, et al.* (2016). Genomic and oncogenic preference of HBV integration in hepatocellular carcinoma. *Nat Commun* **7**: 12992.

14. Xu, M, Yao, Y, Chen, H, Zhang, S, Cao, SM, Zhang, Z, Luo, B, Liu, Z, Li, Z, Xiang, T*, et al.* (2019). Genome sequencing analysis identifies Epstein-Barr virus subtypes associated with high risk of nasopharyngeal carcinoma. *Nat Genet* **51**: 1131-1136.

15. Palser, AL, Grayson, NE, White, RE, Corton, C, Correia, S, Ba Abdullah, MM, Watson, SJ, Cotten, M, Arrand, JR, Murray, PG*, et al.* (2015). Genome diversity of Epstein-Barr virus from multiple tumor types and normal infection. *J Virol* **89**: 5222-5237.

16. Tian, R, Cui, Z, He, D, Tian, X, Gao, Q, Ma, X, Yang, JR, Wu, J, Das, BC, Severinov, K*, et al.* (2019). Risk stratification of cervical lesions using capture sequencing and machine learning method based on HPV and human integrated genomic profiles. *Carcinogenesis*.
